# Effects of beta-blocker withdrawal in patients with heart failure with preserved ejection fraction: A protocol for systematic review and meta-analysis

**DOI:** 10.1371/journal.pone.0294347

**Published:** 2023-11-16

**Authors:** Hidekatsu Fukuta, Toshihiko Goto, Takeshi Kamiya

**Affiliations:** 1 Core Laboratory, Nagoya City University Graduate School of Medical Sciences, Nagoya, Japan; 2 Department of Cardiology, Nagoya City University Graduate School of Medical Sciences, Nagoya, Japan; 3 Department of Medical Innovation, Nagoya City University Graduate School of Medical Sciences, Nagoya, Japan; University of Dundee, UNITED KINGDOM

## Abstract

**Background:**

The primary chronic symptom of patients with heart failure with preserved ejection fraction (HFpEF) is severe exercise intolerance. The inability to adequately increase heart rate during exercise (chronotropic incompetence) is commonly present in HFpEF patients and contributes importantly to exercise intolerance in these patients. Since HFpEF patients often have cardiac comorbidities such as hypertension, coronary artery disease, and atrial fibrillation, beta-blockers are frequently prescribed for the treatment of these comorbidities. However, there is a concern that beta-blockers may worsen chronotropic incompetence by slowing heart rate in HFpEF patients and may further exacerbate their symptoms. There are several studies on the effects of beta-blocker withdrawal in HFpEF patients. We aim to perform the systematic review and meta-analysis of studies on the effects of beta-blocker withdrawal in HFpEF patients.

**Methods:**

This meta-analysis will include randomized controlled trials and prospective cohort studies on the effect of beta-blocker withdrawal in HFpEF patients. Information of studies will be collected from PubMed, Web of Science, and Scopus. The primary outcome will be peak oxygen uptake (peak VO_2_). The secondary outcome will be 6-minute walk distance. Other outcomes of interest will be health-related quality of life, plasma BNP levels, and cardiac structure and function.

**Discussion:**

This systematic review and meta-analysis will evaluate whether beta-blocker withdrawal is beneficial for HFpEF patients, providing evidence regarding beta-blocker withdrawal in these patients.

**Trial registration:**

**Systematic review registration:** INPLASY202370066.

## Introduction

Nearly half of patients with heart failure in the community have preserved ejection fraction (EF) [1,2]. The primary chronic symptom of patients with heart failure with preserved EF (HFpEF) is severe exercise intolerance [3]. The inability to adequately increase heart rate during exercise (chronotropic incompetence) is commonly present in HFpEF patients and contributes importantly to exercise intolerance in these patients [4,5].

Since HFpEF patients are likely to have cardiac comorbidities such as hypertension, coronary artery disease (CAD), and atrial fibrillation (AF), beta-blockers are commonly used for the treatment of these comorbidities [6]. However, several meta-analyses of randomized controlled trials (RCTs) have reported no beneficial effect of beta-blockers in HFpEF patients [7,8]. Furthermore, there is a concern that beta-blockers may worsen chronotropic incompetence by slowing heart rate in HFpEF patients and may further exacerbate their symptoms. There are several studies on the effects of beta-blocker withdrawal in HFpEF patients (ClinicalTrials.gov {NCT03871803}[9]; the Japan Registry of Clinical Trials {jRCT1031210030}). One trial reported the beneficial effect of beta-blocker withdrawal on exercise capacity in HFpEF patients [10]. However, in the trial, beta-blocker withdrawal did not significantly improve cardiac structure or function or plasma B-type natriuretic peptide (BNP) levels, an objective marker of HF severity, due in part to limited power. To gain a better understanding of how beta-blocker discontinuation affects outcomes in HFpEF patients, we aim to perform the systematic review and meta-analysis of studies on the effects of beta-blocker withdrawal in these patients.

## Methods

This study has been registered on International Platform of Registered Systematic Review and Meta-analysis Protocols with registration number of INPLASY202370066 (https://www.doi.org; DOI: 10.37766/inplasy2023.7.0066). This protocol for meta-analysis will be performed according to the Preferred Reporting Items for Systematic Review and Meta-analysis Protocols (PRISMA-P) statement [11].

### Search strategy

The electronic databases for literature search will include PubMed, Web of Science, and Scopus. For search of the eligible studies, the following keywords and Medical Subject Heading will be used:

#1 “heart failure with preserved ejection fraction” OR “heart failure with normal ejection fraction” OR “diastolic heart failure”
#2 “exercise capacity” OR “functional capacity” OR “exercise intolerance” OR “oxygen consumption” OR “oxygen uptake” OR “walk distance” OR “walk test”
#3 “quality of life” OR “left ventricular” OR “left ventricle” OR “systolic function” OR “systolic dysfunction” OR “diastolic function” OR “diastolic dysfunction” OR “left atrium” OR “left atrial” OR “natriuretic peptide”
#4 “beta blockers” AND (“withdrawal” OR “discontinuation”)
#5 #1 AND #2 AND #4 (primary and secondary outcomes)
#6 #1 AND #3 AND #4 (other outcomes)

**Fig 1:**
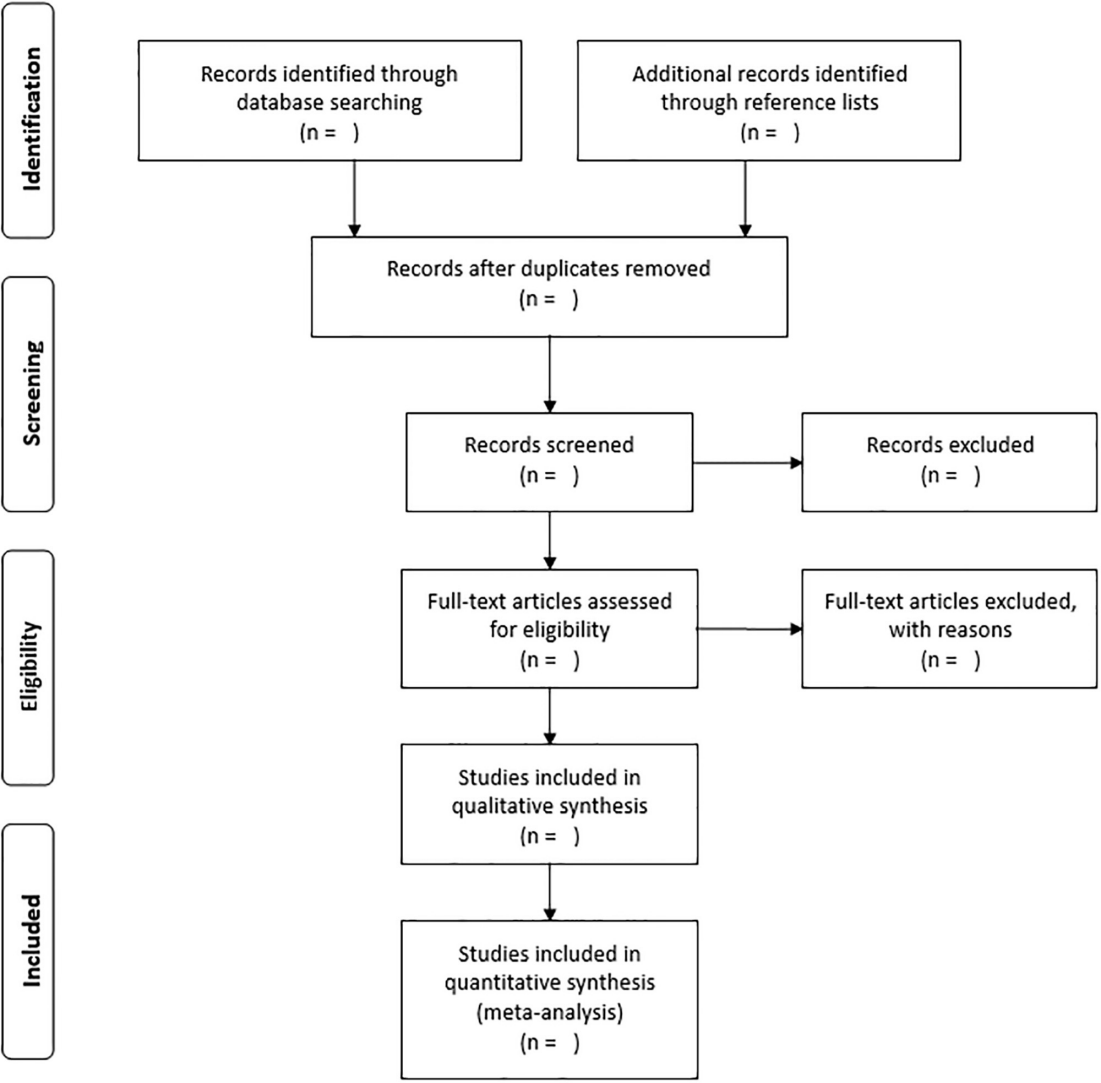
PRISMA flow diagram.

Literature search will also be conducted by manual screening of reference lists of relevant reviews and retrieved articles. Two researchers (HF and TK) will independently perform the literature search and will extract study-level data from included studies. Disagreements will be resolved by consensus. The literature search will be repeated before completing the data extraction, and potential new studies published during the work process will be added to the result. Study selection will be conducted in a PRISMA-compliant flow chart (Fig 1). Only articles published in the English language will be included. The literature search will be started in September 2024 and the data extraction will be completed by December 2024.

### Study design

RCTs and prospective cohort studies will be included. Retrospective cohort and case–control studies will be excluded.

### Selection criteria

Inclusion criteria for this meta-analysis will be: (1) include HFpEF patients treated with beta-blockers; (2) compare between beta-blocker withdrawal and beta-blocker continuation; and (3) assess exercise capacity, health-related quality of life, plasma BNP levels, or cardiac structure or function.

### Outcomes

The primary outcome will be peak oxygen uptake (peak VO_2_). The secondary outcome will be 6-minute walk distance. Other outcomes of interest will be health-related quality of life, plasma BNP levels, and cardiac structure and function. In the measures of health-related quality of life, the scores of the Minnesota Living With Heart Failure Questionnaire, the Kansas City Cardiomyopathy Questionnaire, and the 36-item Short-Form Survey will be extracted. In the measures of cardiac structure, left ventricular (LV) mass and left atrial volume will be extracted. In the measures of LV systolic function, LVEF and early systolic mitral annular velocity (s’) will be extracted. In the measures of LV diastolic function, early diastolic mitral annular velocity (e’) and the ratio of early diastolic mitral inflow to annular velocities (E/e’) will be extracted given the linear relationship with LV diastolic dysfunction grade.

### Data extraction

Two reviewers (HF and TK) will independently extract relevant data from retrieved studies, including author, study design, study time, country, number of participants, baseline characteristics (age, sex, body mass index, comorbidities), clinical outcomes (exercise capacity, health related-quality of life, BNP levels, and cardiac structure and function), and information on the methodological quality (selection of cohorts, assessment of outcome, etc). Disagreements will be resolved by consensus. We will contact the corresponding author of eligible studies when insufficient information is available to perform our meta-analysis.

### Quality assessment

The Cochrane Risk of Bias tool will be used to assess quality of RCTs included[12]. The quality of prospective cohort studies will be evaluated by Newcastle-Ottawa Scale tool (http://www.ohri.ca/programs/clinical_epidemiology/oxford.asp). The quality of evidence for the outcomes will be evaluated by use of the Grading of Recommendations Assessment, Development and Evaluation (GRADE) system [13]. The quality of evidence will be evaluated across the domains of risk of bias, consistency, directness, precision, and publication bias.

### Statistical analysis

For continuous outcomes, the effect size for the intervention will be calculated by the difference between the means of the intervention and control groups at the end of the intervention. If the outcome is measured on the same scale, the weighted mean difference and 95% confidence interval (CI) will be calculated. Otherwise, the standardized mean difference and 95% CI will be calculated. For each outcome, heterogeneity will be assessed using the Cochran’s Q and *I*^2^ statistic; for the Cochran’s Q and *I*^2^ statistic, a p value of <0.1 and *I*^2^>50%, will be considered significant, respectively. When there is significant heterogeneity, the data will be pooled using a random-effects model, otherwise a fixed-effects model will be used. When there are more than 10 studies included, publication bias will be assessed graphically using a funnel plot and mathematically using Egger test. For these analyses, Comprehensive Meta Analysis Software version 2 (Biostat, Englewood, NJ, USA) and STATA 16 software (Stata Corp LP, TX, USA) will be used.

When the number of included studies reporting the primary outcome in the present meta-analysis is less than 3, the above-mentioned pooled analysis will not be conducted. Instead, a systematic narrative synthesis will be provided with information presented in the text and tables to summarize and explain the characteristics and findings of the included studies. The narrative synthesis will explore the relationship and findings both within and between the included studies, in compliance with the guidance from the Centre for Reviews and Dissemination[11].

### Sensitivity analysis

Subgroup analysis stratified by study design (RCT or prospective cohort study) will be performed. Meta-regression will be used to determine whether the effect of beta-blocker withdrawal on exercise capacity will be confounded by baseline clinical characteristics such as age, sex, New York Heart Association functional class, AF, and CAD. Based on an earlier report, when the number of included studies reporting the primary outcome in the present meta-analysis is less than 5, sensitivity analysis will not be performed [14].

### Ethical issues

This meta-analysis is a literature study. Ethical approval is not required because this meta-analysis will not involve any subject directly.

## Discussion

It is well established that beta-blockers decrease LV oxygen consumption requirements by reducing heart rate, systolic blood pressure, and myocardial contractility and thereby improve myocardial ischemia [15]. Additionally, beta-blockers reduce ventricular rate and prolong the diastolic period. Taken together, beta-blockers favorably impact LV diastolic filling by reducing myocardial ischemia and prolonging diastolic period and may thereby improve symptoms and exercise capacity in HFpEF patients, particularly in those with CAD or AF. However, it is possible that beta-blockers may reduce heart rate during exercise (negative chronotropic effect), resulting in unfavorable effect on exercise capacity. Thus, the benefit of beta-blockers on symptoms and exercise capacity in HFpEF patients may depend on the balance between the improved LV diastolic filling and the negative chronotropic effect.

One RCT reported the beneficial effect of beta-blocker withdrawal on exercise capacity in HFpEF patients [10]. However, in the trial, beta-blocker withdrawal did not significantly improve cardiac structure or function or plasma BNP levels, an objective marker of HF severity, due in part to limited power. Furthermore, it should be recognized that the trial was conducted in a selected population and thus the results are not applicable to entire HFpEF population. Given a variety of comorbid conditions of HFpEF patients [6], further studies are necessary to determine which subgroups of HFpEF patients are most likely to benefit from beta-blocker withdrawal.

To the best of our knowledge, this is the first systematic review and meta-analysis protocol on the effect of beta-blocker withdrawal in HFpEF patients. This systematic review and meta-analysis will evaluate whether beta-blocker withdrawal is beneficial for HFpEF patients, providing evidence regarding beta-blocker withdrawal in these patients.

## Supporting information

S1 Checklist PRISMA-P (Preferred Reporting Items for Systematic review and Meta-Analysis Protocols) 2015 checklist: Recommended items to address in a systematic review protocol*. (DOC)

## Abbreviations

AF: atrial fibrillation
BNP: B-type natriuretic peptide
CAD: coronary artery disease
EF: ejection fraction
GRADE: Grading of Recommendations Assessment, Development and Evaluation
HF: heart failure
HFpEF: heart failure with preserved ejection fraction
LV: left ventricular
PRISMA-P: Preferred Reporting Items for Systematic Review and Meta-analysis Protocols
RCTs: randomized controlled trials
